# Evaluating the Carrot Rewards App, a Population-Level Incentive-Based Intervention Promoting Step Counts Across Two Canadian Provinces: Quasi-Experimental Study

**DOI:** 10.2196/mhealth.9912

**Published:** 2018-09-20

**Authors:** Marc Mitchell, Lauren White, Erica Lau, Tricia Leahey, Marc A Adams, Guy Faulkner

**Affiliations:** 1 Western University London, ON Canada; 2 Carrot Insights Inc Toronto, ON Canada; 3 University of British Columbia Vancouver, BC Canada; 4 University of Connecticut Storrs, CT United States; 5 Arizona State University Phoenix, AZ United States

**Keywords:** behavioral economics, financial health incentives, mHealth, mobile phone, physical activity, public health

## Abstract

**Background:**

The Carrot Rewards app was developed as part of an innovative public-private partnership to reward Canadians with loyalty points, exchangeable for retail goods, travel rewards, and groceries for engaging in healthy behaviors such as walking.

**Objective:**

This study examined whether a multicomponent intervention including goal setting, graded tasks, biofeedback, and very small incentives tied to daily step goal achievement (assessed by built-in smartphone accelerometers) could increase physical activity in two Canadian provinces, British Columbia (BC) and Newfoundland and Labrador (NL).

**Methods:**

This 12-week, quasi-experimental (single group pre-post) study included 78,882 participants; 44.39% (35,014/78,882) enrolled in the Carrot Rewards “Steps” walking program during the recruitment period (June 13–July 10, 2016). During the 2-week baseline (or “run-in”) period, we calculated participants’ mean steps per day. Thereafter, participants earned incentives in the form of loyalty points (worth Can $0.04 ) every day they reached their personalized daily step goal (ie, baseline mean+1000 steps=first daily step goal level). Participants earned additional points (Can $0.40) for meeting their step goal 10+ nonconsecutive times in a 14-day period (called a “Step Up Challenge”). Participants could earn up to Can $5.00 during the 12-week evaluation period. Upon meeting the 10-day contingency, participants could increase their daily goal by 500 steps, aiming to gradually increase the daily step number by 3000. Only participants with ≥5 valid days (days with step counts: 1000-40,000) during the baseline period were included in the analysis (n=32,229).The primary study outcome was mean steps per day (by week), analyzed using linear mixed-effects models.

**Results:**

The mean age of 32,229 participants with valid baseline data was 33.7 (SD 11.6) years; 66.11% (21,306/32,229) were female. The mean daily step count at baseline was 6511.22. Over half of users (16,336/32,229, 50.69%) were categorized as “physically inactive,” accumulating <5000 daily steps at baseline. Results from mixed-effects models revealed statistically significant increases in mean daily step counts when comparing baseline with each study week (*P*<.001). Compared with baseline, participants walked 115.70 more steps (95% CI 74.59 to 156.81; *P*<.001) at study week 12. BC and NL users classified as “high engagers” (app engagement above sample median; 15,511/32,229, 48.13%) walked 738.70 (95% CI 673.81 to 803.54; *P*<.001) and 346.00 (95% CI 239.26 to 452.74; *P*<.001) more steps, respectively. Physically inactive, high engagers (7022/32,229, 21.08%) averaged an increase of 1224.66 steps per day (95% CI 1160.69 to 1288.63; *P*<.001). Effect sizes were modest.

**Conclusions:**

Providing very small but immediate rewards for personalized daily step goal achievement as part of a multicomponent intervention increased daily step counts on a population scale, especially for physically inactive individuals and individuals who engaged more with the walking program. Positive effects in both BC and NL provide evidence of replicability.

## Introduction

The health benefits of regular physical activity are unquestionable. Regular moderate-intensity physical activity, brisk walking, for example, reduces the risk of several noncommunicable diseases, such as type 2 diabetes [1,2]. Regular physical activity has also been shown to improve cognition [3], prevent and manage depression [4], and prevent or delay the onset of dementia [5]. Furthermore, a recent analysis of objectively measured physical activity (n=5562 American adults) determined that participation in moderate-intensity physical activity was associated with substantial reduction in mortality risk [6]. For women, even modest participation in *low-intensity* physical activity, for example, slower walking without “huffing and puffing,” was linked with lower mortality risk [6]. Unfortunately, physical inactivity remains a global pandemic [7,8]. Conservative estimates suggest that this pandemic cost the global economy US $53.8 billion in direct health care expenses in 2013 [9]. In Canada, as in most higher-income countries, the public sector bears the largest proportion of health care expenditures attributable to physical inactivity [9].

Behavioral economics, a branch of economics complimented by insights from psychology [10], has stimulated interest in using financial health incentives to promote physical activity [11]. Financial health incentives are defined as rewards with monetary value contingent on achievement of prespecified health behaviors or outcomes [12], such as rewarding people to walk more [13] or to lose weight [14]. One way timely financial incentives might work, according to behavioral economics, is by leveraging people’s predictable tendency to act in favor of their immediate self-interest, a principal referred to as “present bias” [10]. In the case of physical activity, the likelihood that someone will be more physically active should increase if a financial incentive is at stake—and the more immediate the incentive, the stronger the nudge, according to this theoretical perspective [15].

Evidence supporting the use of financial health incentives is growing, with 2 systematic reviews [13,16] and 1 meta-analysis [17] finding that incentives generally increase physical activity in the short-term (≤3 months) and while they are still in place (ie, *before* they are withdrawn). However, evidence regarding *sustained* physical activity increases (ie, *after* incentives are removed) is more mixed, with some randomized controlled trials (RCTs) reporting postintervention benefits [18-21] and others not [22-24]. Finkelstein et al (2016) conducted the largest (N=800) of these trials and found that physical activity was higher among incentive group participants at 6 months, but this effect was not sustained 6 months after incentive removal [24]. The authors suggest that study design (eg, intervention duration), sample characteristics (eg, baseline physical activity), and incentive features (eg, generic, not tailored, physical activity goals) may have moderated postintervention responses. Discrepant findings and a still limited number of studies suggest that more research is needed to elucidate conditions under which incentives are more likely to drive postintervention changes.

In some cases, however, offering incentives for longer periods may be suitable, as Finkelsetin et al (2016) suggest—until a time when physical activity motives are internalized (“I walk because it makes me feel good”) or until clinically meaningful health outcomes are achieved [24]. While acknowledging that more research is needed [25], the 3 RCTs that have tested physical activity incentives for ≥6 months have reported significant, positive effects [22,24,26]. However, the cost of longer term incentive programs may be prohibitive, especially if offered on a population scale. Therefore, at the same time research continues to examine conditions under which incentives drive sustained, long-term changes, efforts to increase efficiency, and thus scalability, of incentive interventions are also needed. The incentive magnitude typically used to promote physical activity in RCT settings (ie, US $1-US $2 per day) [15,19,22,23,27,28] may be simply too high for third-party payers and real-world implementation.

To reduce the cost of incentives and realistically operate within fixed government or insurer budgets, several incentive program features or reinforcement properties can be manipulated (eg, size, immediacy, probability, timing, type of incentive) [11,12,29,30]. For example, by shortening the time between behavior and reward so that rewards are delivered immediately after desired responses, the reward size needed to stimulate physical activity may decrease [11]. Smartphone technology presents an opportunity to provide incentives immediately upon physical activity goal completion (eg, steps per day). Built-in smartphone accelerometers now make it easier to track physical activity (ie, since the Apple Inc. iOS Health Kit app launched in 2014) [31]; furthermore, previously unavailable moment-by-moment physical activity data can now be used to set and personalize physical activity goals and provide immediate feedback in the form of rewards (eg, rewards automatically transmitted to Web-based accounts). Also, loyalty points (ie, points given by retailers to promote customer loyalty) have emerged as a promising new incentive type (vs cash, vouchers, or charity donations) [32-34]. Research shows that consumers tend to overvalue the points they collect (eg, although US $1 cash may have stimulated physical activity in the past, US $0.50 in loyalty points may produce the same effect) [35], possibly lowering the reward size needed to stimulate physical activity. These intervention features (using smartphones to track and reward physical activity with loyalty points) may appeal to governments and insurers looking to deploy financial health incentives more efficiently.

In Canada, such features are now available via the Carrot Rewards app, a new mHealth initiative that rewards Canadians with loyalty points (eg, retail goods, travel, groceries) to engage in healthy behaviors (eg, visiting flu shot clinic, walking) [34,36,37]. This study’s purpose was to examine whether the Carrot Rewards “Steps” walking program, which utilizes very small incentives (Can $ 0.04 in loyalty points) tied to daily step goal achievements could stimulate physical activity in two Canadian provinces.

## Methods

### Background

Carrot Insights Inc. is a private company that developed the free Carrot Rewards app with support from the Public Health Agency of Canada. The British Columbia (BC) Ministry of Health was the company’s founding provincial Ministry partner. Newfoundland and Labrador (NL) was the second Canadian province to offer the app to its residents. Carrot Rewards was made available for BC and NL residents on the Apple iTunes and Google Play app stores on March 3 and June 13, 2016, respectively, in both English and French (Canada’s official languages). Upon downloading the app, the users were asked to enter their age, gender, postal code, and loyalty program card number to complete registration (users without loyalty cards were directed to an easy sign-up page). To register successfully, users must have entered a valid BC or NL postal code and have been ≥13 years (age cutoff of participating loyalty programs). The walking program was not initially available in BC, but was introduced the day the app launched in NL. Carrot Insights Inc. partnered with 4 major Canadian loyalty programs to offer a variety of popular incentives (ie, points could be redeemed for groceries, travel, movies, or gas). While BC users could earn points via any of the 4 participating loyalty programs, NL users could earn points only for the 2 loyalty programs with a regional presence (ie, movies and travel). In addition to the 4 participating loyalty programs, Carrot Insights Inc. also partnered with 4 Canadian health charities (ie, Heart and Stroke Foundation of Canada, Diabetes Canada, Young Men’s Christian Association Canada, and the BC Healthy Living Alliance), primarily for the purpose of reviewing and approving health education content offered in the app. The Behavioural Research Ethics Board of the University of British Columbia approved this study (UBC BREB Number H17-02814).

### Recruitment

The marketing assets of the 4 loyalty programs and 1 charity partner were leveraged so that in the first few weeks, partners could heavily promote the app in both provinces (ie, in BC, partners sent 1.64 million emails to their loyalty members; in NL, the number of emails is unknown). The users were not automatically enrolled in the walking program, but were rather asked to opt-in. Study recruitment was open for approximately 1 month from June 13 to July 10, 2016. To participate, users had to agree to allow the app to access step data tracked and stored in their smartphones and were rewarded Can $0.60 in loyalty points for doing so.

### Study Participants and Design

Registered users from BC (n=65,414) and NL (n=13,468) were eligible to participate in the walking program. However, only those with iPhone version 5S or higher could participate (ie, the Health Kit app, step data aggregator, is supported and preinstalled on these devices). Android smartphone users could also participate, but they were required to download the Health Kit equivalent (ie, Google Fit app) first. Only those who enabled the walking program on their smartphones (ie, allowed the app to access their data) received the intervention. From June 13 to July 10, 2016, 78,882 users from two Canadian provinces (BC and NL) were eligible to participate in the walking program, and 44.39% (35,014/78,882) ultimately activated it on their smartphones during the recruitment period. To examine the effect of this multicomponent intervention on objectively measured daily step counts, a 12-week quasi-experimental (single group pre-post) study design was employed. Testing the walking program simultaneously in 2 provinces provided a direct replication condition.

### Theoretical Underpinnings

This intervention was theoretically based on principles from behavioral economics and self-determination theory. While behavioral economics describes how incentives exploit “present bias” to *stimulate* behaviors [10], self-determination theory focuses on the extent to which behaviors are controlled by external agents (eg, physicians) or contingencies (eg, incentives) and can be *sustained* [38]. A more thorough review of how these theories complement each other in a financial health incentive context is presented elsewhere [39]. Briefly, timely in-app notifications (“Congrats! You have achieved your 6600 daily step goal!”), very small incentives (not to be overly controlling and to protect autonomy), and a personalized approach to goal setting (realistic daily step goals, so users experience success early) were deployed to maintain fidelity to both behavioral economics and self-determination theory. As well, a range of behavior change techniques [40] are embedded in the app, including goal setting, self-monitoring, and biofeedback (ie, feedback using an external monitoring device), and graded tasks (ie, set at “easy” and then their difficulty increased).

### Baseline Period

For a personalized walking goal to be generated (ie, steps per day), users must have accumulated at least 5 valid days during the initial 14-day baseline or “run-in” period. A valid day was defined as any day with step counts from 1000 to 40,000, as these numbers were considered reasonable, not outliers [41]. Days with step counts <1000 were considered days smartphones were not worn, and days with step counts above 40,000 were deemed suspiciously high (eg, technology bug) and were excluded. For users with at least 5 valid days, a daily step count average was calculated for the baseline period, and 1000 steps were added to set the first daily step goal (rounded to the nearest 100 steps). If users did not have a sufficient number of valid days (ie, ≤4 days) during the baseline period, a generic 5000 daily step goal was provided and they were excluded from analysis. The approximate the number of steps taken daily by the average Canadian adult is 5000, as measured by a popular smartphone-based activity tracking app [42].

### Program

**Figure 1:**
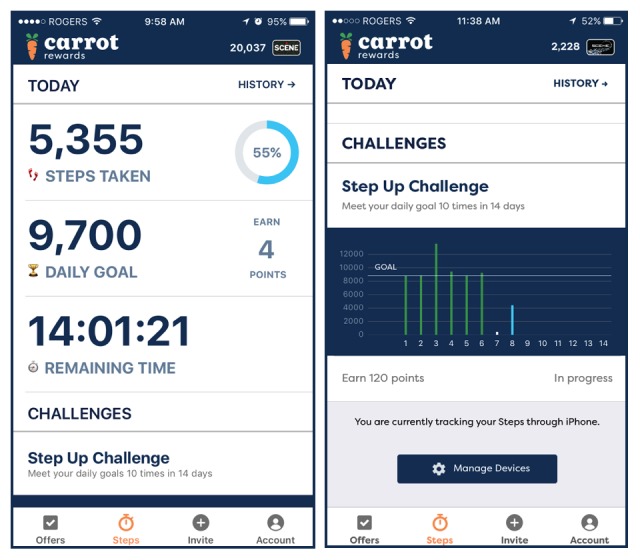
Carrot Rewards app’s “Steps” walking program screenshots.

After the 14-day baseline period, users could begin to earn incentives for reaching or exceeding their individualized daily step goals; a progress wheel illustrated progress for the day (see Figure 1 for walking program screenshots). Incentives for daily achievements were worth Can $0.04 in loyalty points. After 2 weeks of earning daily rewards in the form of points, users could then begin to earn bonus rewards worth Can $0.40 in points for reaching their daily goal ≥10 nonconsecutive times within a 14-day period, called a “Step Up Challenge.” Incentives for longer term (eg, biweekly) physical activity goals, in addition to daily goals only, have worked well in past studies [24]. Users were automatically enrolled in the first “Step Up Challenge,” but thereafter always had to accept the challenge when it became available. A bar graph to illustrate “Step Up Challenge” progress was also made available upon tapping “Accept” in the app (see Figure 1). For users who successfully completed the “Step Up Challenge,” a new higher daily step goal was provided (ie, 500 steps more than the previous goal). For unsuccessful users, the previous goal persisted. Over the 3-month evaluation period, participants could earn a total of Can $5.00 in points (Can $0.60 for activating the walking program, Can $2.80 for daily step goal achievements, and Can $1.60 for successfully completing 4 “Step Up Challenges”).

### Outcome Measures

The primary outcome variable was mean daily step counts as measured by either built-in smartphone accelerometers, for example, iPhone 5S or higher for 53.63% (42,304/78,882) of users, Android devices for 37.48% (29,565/78,882) of users, or any Fitbit device for 7.18% (5664/78,882) of users. Recent validation studies found that the iPhone step counting feature (version 6 or newer), as well as those for Android smartphones (eg, HTC, Motorola) and Fitbit trackers (eg, hip-worn Zip, wrist-worn Flex) were accurate in laboratory and field conditions [43-45]. However, Duncan et al (2018) did determine that steps were underestimated by the iPhone step counting feature in their free-living condition by approximately 1340 steps per day [43]. According to the study authors, this likely reflects not carrying the iPhone continually throughout the day rather than inaccuracy in the step counting feature; they suggest that if adherence can be optimized, smartphones may be suitable for physical activity evaluations.

### Covariates

The majority of demographic variables used to describe the study sample were self-reported (eg, age, gender, province). Median personal income was inferred by linking user postal codes with census data (ie, 2011 National Household Survey) at the local health area level (89) in BC and regional health authority level (4) in NL.

### Data Analyses

Three different analytical approaches were used to account for missing data and to test the sensitivity of our assumptions with the analytical sample: (1) The “any” data approach included participants with valid baseline data (≥5 days in acceptable range during the 14-day baseline period) and at least 1 other valid week (ie, at least 4 valid days in a 7-day week) from study week 1 to 12 (32,229/35,019, 92.03% of those enabling the walking program met these criteria); (2) the “completer” approach included just participants with valid data at baseline and study week 12 (19,964/32,229, 61.94%); and (3) the “imputed” approach included participants with valid baseline data, but no valid data at study week 12 (29,261/32,229, 90.79%). Then, we imputed participants’ “Pseudo study week 12” by carrying forward their baseline values. Therefore, among those included in the analysis (n=32,229), 61.94% (19,964/32,229) had complete datasets (completers). No differences were observed in demographic characteristics between completers and noncompleters (see Table 1). Since the 3 different analytic approaches yielded very similar results, given the public health nature of the intervention and that completers did not differ from noncompleters on key demographic characteristics, analyses using the “any” data approach are presented.

Statistical analysis was performed using R 3.3.0.68 Mavericks build (7202) Rstudio Version 1.0.136 (RStudio, Boston, MA, USA). Study week was treated as a categorical variable (baseline=0, study week 1=1, ..., study week 12=12) to allow for the nonlinear trajectory of daily step counts. Also, the estimate for each study week helped refine the program to maintain user engagement. Mixed-effects models were performed to examine whether there were significant changes in mean daily step counts between baseline and study week 12. We fitted a simple linear mixed-effects model that included study week as the independent variable (baseline data were used as the reference), followed by an adjusted model with random intercepts to account for measurements nesting within individuals and by controlling for age, gender, median personal income, and province as covariates. Analyses were performed on the entire sample, and participants were stratified by physical activity status as defined by Tudor-Locke et al [46] (ie, physically inactive: baseline mean steps per day<5000; physically active: baseline mean steps per day≥5000) and by province (ie, BC and NL).

As suggested by previous studies [47], we examined whether participants’ engagement levels had a moderating effect on intervention outcome. Two additional variables, engagement and study week × engagement, were tested in all models. Engagement was a variable dichotomizing all participants into 2 categories, “high” or “low” engagers, based on the median percentage of days when a “Step Up Challenge” was accepted. The interaction term allows the difference between high and low engagers to differ at baseline and study week 12, while controlling for their baseline values and other covariates. Cohen f^2^ for local effect sizes of mean daily step counts within mixed-effects models were calculated, with f^2^≥0.02, f^2^≥0.15, and f^2^≥0.35 representing small, medium, and large effect sizes, respectively [48]. Least-square means along with *P* values were obtained from mixed-effects models for comparing mean daily step counts between subgroups. All data were expressed in least-square means with 95% CIs. Statistical significance levels were set at *P*<.05.

**Table 1:** Baseline characteristics of Carrot Rewards users, by completion status, and for the general Canadian population.

Characteristics	Completers^a^ (n=19,964)	Noncompleters^b^ (n=12,265)	Canadian population (N=35,151,728)
Age in years, mean (SD)	33.8 (11.4)	33.5 (11.9)	40.6 (median)
Gender (% female)	66.1	66.1	50.4
Province (% British Columbia)	72.1	70.3	13.2
Median personal income (Can $1000/year), mean (SD)	29.7 (4.1)	29.6 (4.0)	33.9
Steps per day, baseline mean (SD)	6665.6 (4220.7)	6157.5 (4388.9)	N/A^c^
Engagement^d^ (% high)	59.4	19.8	N/A

^a^Participants with valid data at baseline and study week 12.

^b^Participants with valid data at baseline, but not at study week 12.

^c^N/A: not applicable.

^d^A variable dichotomizing participants into 2 categories, “high” or “low” engagers, based on the median percentage of days when a “Step Up Challenge” was accepted.

## Results

### Baseline Characteristics

The mean age of the 32,229 participants with valid baseline data was 33.7 (SD 11.6) years; 66.11% (21,306/32,229) were female (Table 1). Participants from BC made up 71.41% (23,016/32,229) of the study sample owing to the province’s larger population and to the app launching 3 months prior to its launch in NL. The mean personal median income was Can $29,650, slightly lower than that of 2014 BC and NL means of Can $31,610 and Can $30,450, respectively [49]. The mean daily step count at baseline was 6511.22 steps per day. Just over half of users 50.69% (16,336/32,229) were categorized as “physically inactive,” having accumulated <5000 daily steps at baseline. Assuming age, income, and province were held constant, male participants walked 2297.50 steps more steps per day at baseline compared with females (*P*<.001), and participants from NL walked 992.95 fewer steps per day than those from BC (*P*<.001).

### Weekly Means

**Figure 2:**
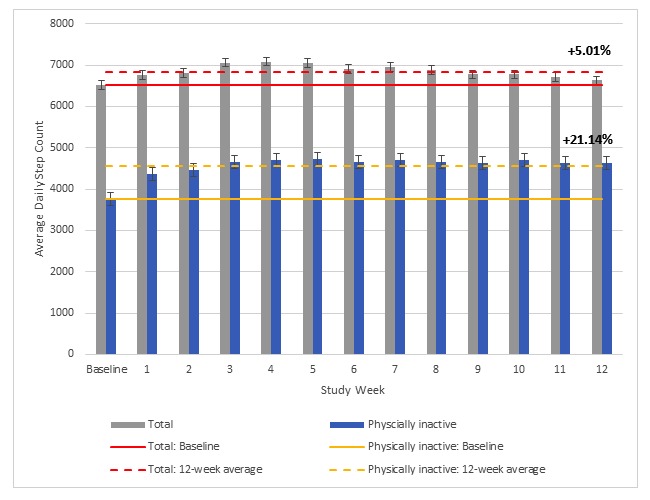
Least-square means for daily steps at baseline and for each study week during the 12-week evaluation period for the total sample and physically inactive participants.

The trends of daily step counts for the total group and the physically inactive subgroup over the 12-week intervention period are illustrated in Figure 2. The difference between baseline and the 12-week evaluation period average for the total group (5.01%) and physically inactive participants (21.14%) are also illustrated. Error bars show 95% CIs. For the total, some behavioral decay was observed in later weeks as the weekly steps per day average dropped below the 12-week intervention mean (6864.77 steps) in study weeks 9 (6772.68 steps) through 12 (6626.92 steps). The average increase in daily step counts over the 12-week intervention period was 353.56 steps, which represents a 5.01% difference from baseline. Among physically inactive users, an average increase of 861.12 steps per day was observed, representing a 21.14% difference from baseline. There was no evidence of behavioral decay in this subgroup as weekly steps per day persisted at or above the intervention mean (4621.76 steps) in study weeks 9 (4622.22 steps) to 12 (4634.83 steps).

**Table 2:** Changes in mean daily step counts between baseline and study week 12.

Analysis			Baseline least-square means^a^ (95% CIs)	Week 12 least-square means^a^ (95% CIs)	Differences (Week 12 – baseline) least-square means^a^ (95% CIs)	Cohen f^2b^
Total sample analysis			6511.22 (6242.24 to 6780.19)	6626.92 (6357.34 to 6896.50)	115.70 (74.59 to 156.81)	0.0059
**Subgroup analyses**						
	**Physical activity status**					
		Physically inactive	3760.64 (3543.31 to 3977.96)	4634.83 (4416.56 to 4853.09)	874.19 (827.98 to 920.40)	0.0234
		Physically active	8778.01 (8392.20 to 9163.81)	8297.19 (7910.38 to 8684.00)	−480.82 (−545.17 to −416.46)	0.0073
	**Province**					
		British Columbia	7064.82 (6796.12 to 7333.52)	7282.83 (7013.40 to 7552.26)	218.01 (169.56 to 266.46)	0.0061
		Newfoundland and Labrador	6071.87 (5790.32 to 6353.43)	5938.22 (5654.07 to 6222.37)	−133.66^c^ (−155.98 to −3.37)	0.0087
	**Engagement**					
		Low engager	6229.27 (5958.75 to 6497.80)	5738.52 (5466.67 to 6010.37)	−490.75 (−551.21 to −428.29	0.0073
		High engager	6780.37 (6509.93 to 7050.81)	7411.27 (7140.55 to 7681.99)	630.90 (575.43 to 686.36)	N/A^d^
		Physically inactive, low engager	3650.63 (3432.54 to 3868.72)	4132.55 (3 911.07 to 4354.04)	481.92 (414.62 to 549.22)	0.0055
		Physically inactive, high engager	3838.79 (3628.93 to 4068.65)	5073.45 (4853.31 to 5293.59)	1224.66 (1160.69 to 1288.63)	N/A
		Physically active, low engager	8588.00 (8200.39 to 8975.61)	7258.26 (6866.58 to 7649.93)	−1329.74 (−1427.93 to −1231.56)	0.0096
		Physically active, high engager	8928.63 (8540.95 to 9316.31)	9131.09 (8742.81 to 9519.36)	202.26 (117.20 to 287.72)	N/A
		British Columbia, low engager	6771.26 (6500.00 to 7042.52)	6353.11 (6078.918 to 6627.31)	−418.15 (−491.12 to −345.17)	0.0071
		British Columbia, high engager	7316.68 (7044.75 to 7588.61)	8055.38 (7783.15 to 8327.61)	738.70 (673.81 to 803.54)	N/A
		Newfoundland and Labrador, low engager	5769.62 (5480.24 to 6059.00)	5120.22 (4822.23 to 5418.21)	−649.40 (−763.50 to −535.30)	0.0074
		Newfoundland and Labrador, high engager	6369.92 (6072.63 to 6667.21)	6715.92 (6417.20 to 7014.64)	346.00 (239.26 to 452.74)	N/A

^a^Least-square means adjusted for age, median personal income, gender, and province.

^b^Cohen f^2^≥0.02, ≥0.15, and ≥0.35 representing small, medium, and large effect sizes, respectively. For the engagement subgroup analysis only, Cohen f^2^ was calculated for the pre-post difference in steps between the low and high engagement groups (high engagement as the referent group).

^c^The difference between baseline and week 12 were statistically significant at *P*<.001 for total sample and all subgroup analyses, except for Province Newfoundland and Labrador (*P*<.001).

^d^N/A: not applicable.

### Total Sample Analysis

The results from mixed-effects models revealed statistically significant increases in mean daily step counts when comparing baseline with each study week (*P*<.001). Changes in mean daily step count from baseline to study week 12 expressed in least-square means are presented in Table 2. Overall, compared with baseline, participants walked 115.70 more steps (95% CI 74.59 to 156.81; *P*<.001) at study week 12. The Cohen f^2^ value was 0.0059 (*P*<.001), indicating the effect was modest. Adjusting for demographic variables (ie, age, gender, province, and median personal income) had little effect on the estimated difference between study week 12 and baseline.

### Subgroup Analysis

The intervention effect was more pronounced in physically inactive users than in physically active users. As with the total sample analysis, the mean daily steps were significantly higher for physically inactive users at each study week than at baseline (*P*<.001), with an observed increase of 874.19 steps per day at study week 12 (Table 2; 95% CI 827.98 to 920.40, *P*<.001). Cohen f^2^ statistic indicated that the effect was small (0.0234, *P*<.001). At study week 12, compared with baseline, a highly significant decrease of 480.82 steps per day was observed among physically active participants (Table 2; 95% CI −545.17 to −416.46, *P*<.001, Cohen f^2^=0.0073, *P*<.001). Participants from NL did not respond as well as participants from BC. At study week 12, compared with baseline, a highly significant increase of 218.01 was observed in BC (Table 2; 95% CI 169.56 to 266.46, *P*<.001, Cohen f^2^=0.0061, *P*<.001), while a highly significant decrease of 133.66 steps per day was observed in NL (Table 2; 95% CI −155.98 to −3.37, *P*<.001, Cohen f^2^=0.0087, *P*<.001).

### Moderation Analysis

Participant engagement showed a significant moderating effect on the intervention outcome in all models (*P*<.001). Therefore, we also conducted subgroup analysis by participants’ engagement levels. As shown in Table 2, all subgroups except physically active low engagers showed significant increase in step counts from baseline to study week 12. The difference from baseline to study week 12 for high (15,511/32,229; 48.13%) and low engagers (16,718/32,229; 51.87%) was +630.90 and −489.75 steps per day, respectively (*P*<.001). As well, users classified as high engagers in BC and NL walked 738.70 and 346.00 more steps per day, respectively (*P*<.001). Among users classified as high engagers and physically inactive (7,022/32,229; 21.08%), an average increase of 1224.66 steps per day was observed (*P*<.001).

## Discussion

### Principal Findings

In this large quasi-experimental study examining the impact of a multicomponent intervention on objectively measured daily step count, a small but significant effect overall was observed (5% average daily step count increase over 12 weeks vs baseline) with a more pronounced effect (21% increase) among physically inactive users (representing over half of the total sample). Notably, this effect was evident irrespective of age, gender, or median personal income. While the overall effect was small (ie, 116 steps per day), these results underscore the potential public health impact of using modest incentives (Can $ 0.04 per day) to stimulate physical activity, particularly among higher risk, physically inactive populations. When considering the clinical significance of this study’s results, it is likely that health benefits (eg, better glucose control) [1] might be reserved for 51% of the analytic sample that increased their daily step counts by 874 steps per day (the physically inactive). Health economic implications of initiatives like this may be important, especially considering that a mere 1% reduction in the number of Canadians classified as physically inactive would yield annual health care savings of Can $2.1 billion [50]. The combination of immediate rewards in the form of loyalty points tied to smartphone-assessed physical activity outcomes may prove an efficient way of delivering financial health incentives while still producing a measurable effect.

Other reinforcement-based methods of increasing health behaviors have included using deposit contracts (ie, participants wager their own money) [51], chance-based designs (ie, 1 in 3 chance of earning Can $3 vs just Can $1 per day) [52] and loss-framing (ie, incentive given up front and then *taken away* if goal unmet) [23]. While deposit contract, chance-based and loss-framed designs may be effective, they may also limit enrollment (in the case of deposits) and may be less palatable to governments or insurers looking to deploy such programs (eg, raising concerns about gambling or punishing citizens or employees for not meeting health goals) [53]. This study provides evidence that even very small incentives, as modest as Can $0.04 per day, can be implemented as part of a multicomponent intervention and on a population scale to increase walking and other ambulatory behaviors effectively.

### Attrition

Behavioral decay (ie, steps per day decline) was noted as time passed, with weekly steps per day averages dropping below the intervention mean in later weeks. While this was observed in the total sample (driven by the 480.82 daily step count *reduction* among physically active users), step counts persisted throughout the 12-week evaluation period in the physically inactive subgroup. At study week 12, for example, physically inactive participants were walking 874.19 more steps per day on average (vs baseline). This is consistent with incentives for physical activity literature that suggests that physically inactive adults are more sensitive to incentive interventions and more likely to sustain the behavior for longer periods [17]. Similarly, larger intervention effect sizes are observed among insufficiently active individuals in Web-based physical activity interventions [54]. Why daily step counts *decreased* among physically active participants remains unclear. Seasonal effects may partly explain the drop (the evaluation period began in warmer spring and summer seasons and ended in the colder fall). Smartphone (ie, accelerometer) wear time may also explain the decrease. Physically active users, being generally less sensitive to physical activity incentives, may have carried their smartphones less and less (and recorded fewer and fewer steps) as the intervention progressed.

### Provincial Differences

Regarding provincial differences, NL users did not respond as well as BC users (−133.66 steps per day vs +218.01 steps per day at study week 12, respectively). This could be due to a number of factors. The most important factor may have to do with the walking program’s availability to *all* NL participants right away (upon downloading the app), while BC users who were still engaging with the app 3 months after it launched could activate the walking program (self-selection bias). Additionally, these provinces are on opposite Canadian coasts, with distinct climates and chronic disease risk profiles. Regarding climate, in the final 3 weeks of the evaluation period (when the provincial step count disparity was greatest, ending on October 17, 2016), residents of St. John’s, NL, experienced more “cold days” (ie, below our operational 13.0°C threshold) than their Vancouver, BC, counterparts; 43% (10/23) versus 13% (3/23) of days were “cold”; St. John’s and Vancouver are the largest cities in NL and BC, respectively). Regarding chronic disease risk, while BC has the lowest self-reported adult overweight and obesity rate in Canada (48.0%), NL has the highest (67.5%). Notably, while NL users in general experienced a 133.66 step per day decrease (with low engagers experiencing an even greater 649.40 steps per day drop), a 346.00 step per day increase was observed at study week 12 (vs baseline) among high engagers (3846/9209, 37.85% of the provincial sample). App engagement therefore appears to have boosted intervention effectiveness, regardless of province, suggesting potential effect replication in other jurisdictions. This aligns with broader evidence that greater engagement with a physical activity app or website is associated with increased intervention efficacy [55]. Developing innovative strategies to increase and maintain engagement is a priority (eg, machine learning informed push notifications when “Step Up Challenge” was not accepted within 3 days, rewards for just accepting challenges, small team-based challenges).

### Limitations

The results of this population-level study should be interpreted with caution because there are a number of limitations to consider. First, neither the randomization of participants into intervention and control groups was logistically feasible within this quasi-experimental design nor was the identification of a nonequivalent control group (ie, a group not randomly assigned to receive or not receive the intervention) [56]. For this reason, internal validity (ie, the extent to which causality can be established) may be limited. To improve internal validity as much as possible in this real-world setting, we sought to define a time period that reflected the counter-factual (ie, outcome if the intervention had not been implemented) [56]. To do this, a preintervention time period clearly differentiated from the intervention was introduced. An immediate increase in daily step count compared with baseline was expected, and this is what was observed. This increase, however, may have occurred because participants simply started carrying their smartphones more (the most likely alternative explanation or rival hypothesis) to get credit for the steps they were taking. Disentangling “wear time” from increased actual daily step count is difficult, however, a limitation cited in more carefully controlled RCTs [24]. Additionally, more smartphone accelerometer validation studies are likely required in free-living conditions and with different demographic groups to increase confidence in results. Analysis-phase strategies were employed to improve internal validity as well, including (a) testing the sensitivity of assumptions made with 3 different analytic samples to handle missing data and (b) fitting an adjusted mixed-effects model to account for measurements nesting within individuals and controlling for key demographics. As well, an increase in steps in high, but not low, engagers provides further support for the main conclusion that this multicomponent intervention, when utilized above a threshold level, appears to have yielded daily step count improvements. That behavioral decay was noted in weeks 9-12 for the total sample, but not for the physically inactive subgroup (the group more likely to respond to an incentive-based intervention with realistic and personalized goals) also suggests that the intervention achieved its intended effect of stimulating physical activity among the least active. While traditional RCTs strongly prioritize internal validity, this quasi-experimental design seeks to achieve greater balance between internal and external validity in real-world conditions to facilitate real-world implementation. A second limitation was that participants were followed for only 12 weeks, so longitudinal work is required to elucidate longer term effects. Third, this analysis addressed only the earliest Carrot Rewards app adopters and includes just Canadian provinces, so results may not be generalizable to newer users or other countries. Next, only 44.39% (35,014/78,882) of eligible users who could enable the walking program and earn additional incentives did so during the 4-week recruitment period. How those who activated the program during the recruitment period compare with those who did not remains unknown. While on a population scale this recruitment rate is impressive, there is room to improve. The less than ideal recruitment rate may be because health app users in general discontinue use within days or weeks of first download [47] or a too-short recruitment period. Lastly, at what intensity any extra walking may have occurred is unknown. The association between physical activity and key health outcomes (eg, cardiovascular disease risk factor reduction) is stronger with higher intensity physical activities [6].

### Future Research

To increase internal validity in this quasi-experimental environment, future studies might incorporate interrupted time series, stepped-wedge, intervention removal, or designs with a nonequivalent control group [56]. Future work might also compare different ways of setting and graduating daily step goals (eg, static vs adaptive goal setting) and include longitudinal analyses examining longer term (at least 6 months) impacts, as well as associated cost-effectiveness studies. For example, an adaptive goal setting feature was introduced in the app in February 2017 (after the study period), when step goals began to be recalculated every 2-4 weeks to encourage engagement (as opposed to the “set it and forget it” approach initially adopted). Examining alternative methods to promote sustained physical activity should continue to be a priority for researchers and others in this field (eg, moving from small, regularly scheduled incentives, to large, more irregular, and less predictable ones). To increase the chances of behavior maintenance, exploring opportunities for enhanced engagement that also promote social interaction and support could be a particular focus of future work (eg, encouraging social networking).

### Conclusions

Until recently, financial health incentive programs have shown promise, but little potential for scalability given rewards’ cost. This study adds to the understanding of how incentives can be delivered in ways that are not prohibitively costly. Providing immediate rewards for personalized daily step goal achievement as part of a multicomponent intervention appears to have increased daily step counts on a population scale, especially for higher risk, physically inactive individuals. Positive effects in both BC and NL provide evidence of replicability.

## Abbreviations

BC: British Columbia
NL: Newfoundland and Labrador
RCT: randomized controlled trials

## References

[1] Lee I, Shiroma EJ, Lobelo F, Puska P, Blair SN, Katzmarzyk PT, Lancet PASWG (2012). Effect of physical inactivity on major non-communicable diseases worldwide: an analysis of burden of disease and life expectancy. Lancet.

[2] Warburton DE, Charlesworth S, Ivey A, Nettlefold L, Bredin SS (2010). A systematic review of the evidence for Canada's Physical Activity Guidelines for Adults. Int J Behav Nutr Phys Act.

[3] Khan NA, Hillman CH (2014). The relation of childhood physical activity and aerobic fitness to brain function and cognition: a review. Pediatr Exerc Sci.

[4] Mammen G, Faulkner G (2013). Physical activity and the prevention of depression: a systematic review of prospective studies. Am J Prev Med.

[5] Blondell SJ, Hammersley-Mather R, Veerman JL (2014). Does physical activity prevent cognitive decline and dementia?: A systematic review and meta-analysis of longitudinal studies. BMC Public Health.

[6] Borgundvaag E, Janssen I (2017). Objectively Measured Physical Activity and Mortality Risk Among American Adults. Am J Prev Med.

[7] Kohl HW, Craig CL, Lambert EV, Inoue S, Alkandari JR, Leetongin G, Kahlmeier S, Lancet PASWG (2012). The pandemic of physical inactivity: global action for public health. Lancet.

[8] Sallis JF, Bull F, Guthold R, Heath GW, Inoue S, Kelly P, Oyeyemi AL, Perez LG, Richards J, Hallal PC, Lancet PAS2EC (2016). Progress in physical activity over the Olympic quadrennium. Lancet.

[9] Ding D, Lawson KD, Kolbe-Alexander TL, Finkelstein EA, Katzmarzyk PT, van MW, Pratt M, Lancet PAS2EC (2016). The economic burden of physical inactivity: a global analysis of major non-communicable diseases. Lancet.

[10] Camerer C, Loewenstein G (2003). Behavioral Economics: Past, Present, Future. Advances in Behavioral Economics.

[11] Loewenstein G, Asch DA, Volpp KG (2013). Behavioral economics holds potential to deliver better results for patients, insurers, and employers. Health Aff (Millwood).

[12] Adams J, Giles EL, McColl E, Sniehotta FF (2014). Carrots, sticks and health behaviours: a framework for documenting the complexity of financial incentive interventions to change health behaviours. Health Psychol Rev.

[13] Strohacker K, Galarraga O, Williams DM (2014). The impact of incentives on exercise behavior: a systematic review of randomized controlled trials. Ann Behav Med.

[14] Burns RJ, Donovan AS, Ackermann RT, Finch EA, Rothman AJ, Jeffery RW (2012). A theoretically grounded systematic review of material incentives for weight loss: implications for interventions. Ann Behav Med.

[15] Adams MA, Hurley JC, Todd M, Bhuiyan N, Jarrett CL, Tucker WJ, Hollingshead KE, Angadi SS (2017). Erratum to: Adaptive goal setting and financial incentives: a 2 × 2 factorial randomized controlled trial to increase adults' physical activity. BMC Public Health.

[16] Barte JCM, Wendel-Vos GCW (2017). A Systematic Review of Financial Incentives for Physical Activity: The Effects on Physical Activity and Related Outcomes. Behav Med.

[17] Mitchell MS, Goodman JM, Alter DA, John LK, Oh PI, Pakosh MT, Faulkner GE (2013). Financial incentives for exercise adherence in adults: systematic review and meta-analysis. Am J Prev Med.

[18] Charness G (2009). Incentives to exercise. Econometrica.

[19] Kullgren JT, Harkins KA, Bellamy SL, Gonzales A, Tao Y, Zhu J, Volpp KG, Asch DA, Heisler M, Karlawish J (2014). A mixed-methods randomized controlled trial of financial incentives and peer networks to promote walking among older adults. Health Educ Behav.

[20] Patel MS, Asch DA, Rosin R, Small DS, Bellamy SL, Eberbach K, Walters KJ, Haff N, Lee SM, Wesby L, Hoffer K, Shuttleworth D, Taylor DH, Hilbert V, Zhu J, Yang L, Wang X, Volpp KG (2016). Individual Versus Team-Based Financial Incentives to Increase Physical Activity: A Randomized, Controlled Trial. J Gen Intern Med.

[21] Andrade LF, Barry D, Litt MD, Petry NM (2014). Maintaining high activity levels in sedentary adults with a reinforcement-thinning schedule. J Appl Behav Anal.

[22] Pope L, Harvey J (2014). The efficacy of incentives to motivate continued fitness-center attendance in college first-year students: a randomized controlled trial. J Am Coll Health.

[23] Patel MS, Asch DA, Volpp KG (2016). Framing Financial Incentives to Increase Physical Activity Among Overweight and Obese Adults. Ann Intern Med.

[24] Finkelstein EA, Haaland BA, Bilger M, Sahasranaman A, Sloan RA, Nang EEK, Evenson KR (2016). Effectiveness of activity trackers with and without incentives to increase physical activity (TRIPPA): a randomised controlled trial. Lancet Diabetes Endocrinol.

[25] Mantzari E, Vogt F, Shemilt I, Wei Y, Higgins JPT, Marteau TM (2015). Personal financial incentives for changing habitual health-related behaviors: A systematic review and meta-analysis. Prev Med.

[26] Jeffery RW, French SA (1999). Preventing weight gain in adults: the pound of prevention study. Am J Public Health.

[27] Shin DW, Yun JM, Shin J, Kwon H, Min HY, Joh H, Chung WJ, Park JH, Jung K, Cho B (2017). Enhancing physical activity and reducing obesity through smartcare and financial incentives: A pilot randomized trial. Obesity (Silver Spring).

[28] Kranker K (2018). The Efficacy of Using Financial Incentives to Change Unhealthy Behaviors Among a Rural Chronically Ill and Uninsured Population. Am J Health Promot.

[29] Mitchell MS, Goodman JM, Alter DA, Oh PI, Faulkner GEJ (2015). Development of the Health Incentive Program Questionnaire (HIP-Q) in a cardiac rehabilitation population. Transl Behav Med.

[30] Haff N, Patel MS, Lim R, Zhu J, Troxel AB, Asch DA, Volpp KG (2015). The role of behavioral economic incentive design and demographic characteristics in financial incentive-based approaches to changing health behaviors: a meta-analysis. Am J Health Promot.

[31] Apple (2014). www.apple.com.

[32] Liu S, Hodgson C, Zbib AM, Payne AYM, Nolan RP (2014). The effectiveness of loyalty rewards to promote the use of an Internet-based heart health program. J Med Internet Res.

[33] Goyal S, Morita PP, Picton P, Seto E, Zbib A, Cafazzo JA (2016). Uptake of a Consumer-Focused mHealth Application for the Assessment and Prevention of Heart Disease: The <30 Days Study. JMIR Mhealth Uhealth.

[34] Mitchell M, White L, Oh P, Alter D, Leahey T, Kwan M, Faulkner G (2017). Uptake of an Incentive-Based mHealth App: Process Evaluation of the Carrot Rewards App. JMIR Mhealth Uhealth.

[35] Van Osselaer SM, Alba J, Manchanda P (2004). Irrelevant information and mediated intertemporal choice. Journal of Consumer Psychology.

[36] Government of Canada (2015). www.canada.ca.

[37] Dale LP, White L, Mitchell M, Faulkner G (2018). Smartphone app uses loyalty point incentives and push notifications to encourage influenza vaccine uptake. Vaccine.

[38] Deci E, Ryan R (2002). Handbook of Self-Determination Research.

[39] Mitchell M, Faulkner G (2013). A Nudge at All? The Jury Is Still Out on Financial Health Incentives. Healthcare Papers.

[40] Michie S, Richardson M, Johnston M, Abraham C, Francis J, Hardeman W, Eccles MP, Cane J, Wood CE (2013). The behavior change technique taxonomy (v1) of 93 hierarchically clustered techniques: building an international consensus for the reporting of behavior change interventions. Ann Behav Med.

[41] Bassett DR, Wyatt HR, Thompson H, Peters JC, Hill JO (2010). Pedometer-measured physical activity and health behaviors in U.S. adults. Med Sci Sports Exerc.

[42] Althoff T, Sosič R, Hicks JL, King AC, Delp SL, Leskovec J (2017). Large-scale physical activity data reveal worldwide activity inequality. Nature.

[43] Duncan MJ, Wunderlich K, Zhao Y, Faulkner G (2017). Walk this way: validity evidence of iphone health application step count in laboratory and free-living conditions. J Sports Sci.

[44] Hekler EB, Buman MP, Grieco L, Rosenberger M, Winter SJ, Haskell W, King AC (2015). Validation of Physical Activity Tracking via Android Smartphones Compared to ActiGraph Accelerometer: Laboratory-Based and Free-Living Validation Studies. JMIR Mhealth Uhealth.

[45] Evenson KR, Goto MM, Furberg RD (2015). Systematic review of the validity and reliability of consumer-wearable activity trackers. Int J Behav Nutr Phys Act.

[46] Tudor-Locke C, Craig CL, Thyfault JP, Spence JC (2013). A step-defined sedentary lifestyle index: <5000 steps/day. Appl Physiol Nutr Metab.

[47] Vandelanotte C, Müller AM, Short CE, Hingle M, Nathan N, Williams SL, Lopez ML, Parekh S, Maher CA (2016). Past, Present, and Future of eHealth and mHealth Research to Improve Physical Activity and Dietary Behaviors. J Nutr Educ Behav.

[48] Selya AS, Rose JS, Dierker LC, Hedeker D, Mermelstein RJ (2012). A Practical Guide to Calculating Cohen's f(2), a Measure of Local Effect Size, from PROC MIXED. Front Psychol.

[49] Table17-10-0005-01 Population estimates on July 1st, by age and sex and Table 11-10-0008-01 Tax filers and dependants with income by total income, sex and age database on the Internet.

[50] Krueger H, Turner D, Krueger J, Ready AE (2014). The economic benefits of risk factor reduction in Canada: tobacco smoking, excess weight and physical inactivity. Can J Public Health.

[51] Washington W, McMullen D, Devoto A (2016). A Matched Deposit Contract Intervention to Increase Physical Activity in Underactive and Sedentary Adults. Translational Issues is Psychology Science.

[52] Leahey TM, Subak LL, Fava J, Schembri M, Thomas G, Xu X, Krupel K, Kent K, Boguszewski K, Kumar R, Weinberg B, Wing R (2015). Benefits of adding small financial incentives or optional group meetings to a web-based statewide obesity initiative. Obesity (Silver Spring).

[53] Promberger M, Brown RCH, Ashcroft RE, Marteau TM (2011). Acceptability of financial incentives to improve health outcomes in UK and US samples. J Med Ethics.

[54] Davies CA, Spence JC, Vandelanotte C, Caperchione CM, Mummery WK (2012). Meta-analysis of internet-delivered interventions to increase physical activity levels. Int J Behav Nutr Phys Act.

[55] Schoeppe S, Alley S, Van LW, Bray NA, Williams SL, Duncan MJ, Vandelanotte C (2016). Efficacy of interventions that use apps to improve diet, physical activity and sedentary behaviour: a systematic review. Int J Behav Nutr Phys Act.

[56] Handley MA, Lyles CR, McCulloch C, Cattamanchi A (2018). Selecting and Improving Quasi-Experimental Designs in Effectiveness and Implementation Research. Annu Rev Public Health.

